# Ultrasound Cyclo Plasty for Treatment of Surgery-Naïve Open-Angle Glaucoma Patients: A Prospective, Multicenter, 2-Year Follow-Up Trial

**DOI:** 10.3390/jcm10214982

**Published:** 2021-10-27

**Authors:** Michele Figus, Chiara Posarelli, Marco Nardi, Ingeborg Stalmans, Evelien Vandewalle, Shlomo Melamed, Alon Skaat, Ari Leshno, David Cordeiro Sousa, Luis Abegão Pinto

**Affiliations:** 1 Department of Surgical, Medical, Molecular Pathology and of Critical Area, University of Pisa, 56100 Pisa, Italy; michele.figus@unipi.it (M.F.); marco.nardi@med.unipi.it (M.N.); 2 Department of Ophthalmology, University Hospital of UZ Leuven, 3001 Leuven, Belgium; ingeborg.stalmans@kuleuven.be (I.S.); evelien.vandewalle@kuleuven.be (E.V.); 3 The Sam Rothberg Glaucoma Center, Goldschleger Eye Institute, Sheba Medical Center, Tel-Aviv University, Tel-Hashomer 52621, Israel; melamedshlomo@gmail.com (S.M.); askaat11@gmail.com (A.S.); arileshno@gmail.com (A.L.); 4 Department of Ophthalmology, Santa Maria Hospital, University of Lisbon, 1649-028 Lisbon, Portugal; davidsousa@medicina.ulisboa.it (D.C.S.); abegaopinto@gmail.com (L.A.P.)

**Keywords:** glaucoma, ultrasound, ciliary body, intraocular pressure, prospective study

## Abstract

Background: The purpose of this prospective study was to evaluate the efficacy and safety of the Ultrasound Cyclo Plasty (UCP) procedure using high-intensity focused ultrasound in surgery-naïve open-angle glaucoma patients. Methods: prospective, non-randomized, single-arm, multicenter clinical trial. Sixty-six eyes with primary open-angle glaucoma, intraocular pressure (IOP) ≥21 mmHg and with no history of filtering surgery were enrolled. Patients were treated by UCP with a therapy probe comprising six piezoelectric transducers, consecutively activated for 8 s each. Complete ophthalmic examination was performed before the procedure, 1 day after the procedure, and 1, 3, 6, 12, 18 and 24 months after the procedure. Primary outcomes were complete success (defined as IOP lowering from baseline ≥20% without additional glaucoma medications) and vision-threatening complications. Secondary outcomes were the presence of complications and the reduction of the number of medications used. Results: IOP was significantly reduced after one procedure (*p* < 0.05), from a mean pre-operative value of 24.3 ± 2.9 mmHg (n = 2.3 hypotensive medications) to a mean value of 15.9 ± 3.6 mmHg (n = 2.2 hypotensive medications) at 2 years (mean IOP lowering of 33%). Surgical success was achieved in 74% of eyes. Notwithstanding side effects such as transient anterior chamber inflammation, refractive error changes, transient hypotony and macular edema, no major intra or post-operative complications such as phthisis, induced cataract, neovascularization or significant vision loss were observed. Conclusions: Ultrasound Cyclo Plasty is a valuable, effective and well-tolerated procedure to lower IOP in patients with open-angle glaucoma without previous filtering surgery.

## 1. Introduction

Glaucoma treatment mainly aims to reduce intraocular pressure (IOP), which is the principal risk factor, in order to slow the progress of the disease and preserve the remaining visual acuity, visual function and quality of life at a reasonable cost. The two main therapeutic strategies are either to restore sufficient drainage of the aqueous humor or to reduce the production of aqueous humor. The EyeOP-1 device (EYE TECH CARE—Rillieux-La-Pape—France) is used to reduce the production of aqueous humor via well-controlled thermal coagulation of part of the ciliary body, using high-intensity focused ultrasound (HIFU) [1,2,3,4,5,6].

Several animal studies and subsequent clinical studies have reported that HIFU is effective in terms of IOP lowering in refractory glaucoma (38 to 42%) [7,8,9,10,11,12,13]. Functional in vivo studies have demonstrated the efficacy of coagulation of part of the ciliary body with HIFU. Histological and macroscopic results have shown good local tolerance of the treatment and accurate and targeted coagulation of the ciliary body [14]. Clinical studies have confirmed the tolerance and efficacy of the HIFU procedure. This ultrasound treatment allows significant lowering of IOP (30–40%) without serious complications or major adverse effects [15,16,17,18,19,20,21]. The purpose of this study was to collect safety and efficacy data prospectively for 24 months after the Ultrasound Cyclo Plasty (UCP) treatment using HIFU with the EyeOP-1 in open-angle glaucoma patients without history of filtering surgery.

## 2. Materials and Methods

This was a non-randomized, prospective, single-arm, multicenter follow-up study on surgery-naïve open-angle glaucoma (OAG) patients. The study was conducted according to the principles defined in the Declaration of Helsinki and amendments, was approved by the ethics committee or institutional review board at each site (EC of Leuven: B322201629203; Lisboa EC: 78/16; EC of Tel-Aviv: N° 2917-16-SMC; EC of Pisa: ID 6942) and was registered on ClinicalTrials.gov (NCT02789293).

Patients scheduled for UCP were recruited between April 2017 and December 2017. All patients gave written informed consent. Inclusion criteria were primary open angle glaucoma (POAG), pigmentary glaucoma (PG), or pseudoexfoliative glaucoma (PXG); IOP not adequately controlled with glaucoma medication; IOP ≥21 mmHg and <30 mmHg; no previous intraocular surgery or laser treatment during the 90 days before the UCP procedure; age >18 years and <90 years; able and willing to complete post-operative follow-up requirements. Exclusion criteria were normal tension glaucoma; history of glaucoma surgery failure including trabeculectomy, deep sclerectomy, long-tube drainage devices, cyclo-cryotherapy and cyclophotocoagulation by laser; ocular or retrobulbar tumor; ocular infection within 14 days prior to the UCP procedure; ocular disease other than glaucoma that may affect assessment of visual acuity and/or IOP (including choroidal hemorrhage or detachment, lens subluxation, thyroid ophthalmopathy, proliferative diabetic retinopathy and clinically significant macular edema).

### 2.1. UCP Procedure

The EyeOP-1 device consists of two elements: the control unit, to which a foot pedal is connected, and a sterile, single-use, disposable EyeOP-PACK. The EyeOP-PACK is composed of two parts: a positioning cone and a therapy probe. Probe models, with different ring diameters, equipped with six transducers, are available in three sizes (11 mm, 12 mm and 13 mm). The control unit comprises a generator for power delivery to the therapy probe, a pressure vacuum system enabling the coupling cone to be securely positioned on the eyeball, a touch-screen for setting up treatment parameters and a foot pedal. Each transducer was activated for 8 s at a frequency of 21 MHz (8 s ultrasound, 20 s pause), inducing well-controlled coagulation of the ciliary processes (cyclo-coagulation).

The positioning cone was placed in direct contact with the sclera, which allowed for proper positioning of the transducers in terms of centering and distance. At the base of the cone, a suction ring allowed a low-level vacuum to be applied and enabled the cone to remain in contact with the eye. The probe, containing six active piezoelectric elements, was inserted in the upper part of the positioning cone, leaving a space between the probe and the ocular surface. The six transducers are located at regular intervals on the upper and lower circumference of the ring, avoiding the nasal and temporal meridians, and are oriented to create a focal zone consisting of six regularly distributed elliptical cylinder-shaped volumes. The physician paid particular attention to the horizontal orientation of the probe to ensure that the nasal and temporal sectors were not treated. The cavity created between the eye, the cone and the probe (4 mL) was filled with room temperature saline solution.

In each patient, the probe model whose focal zones matched the ciliary body was determined via ultrasound biomicroscopy (UBM) imaging or Visante optical coherence tomography (OCT) of the anterior segment performed before the procedure, or using anatomic parameters (white-to-white and axial length measurements). UCP was conducted in the operating theater, and locoregional anesthesia (retrobulbar) was performed before the procedure. After UCP, appropriate anti-inflammatory drops (steroid eye-drops for 3–4 weeks) were prescribed. Other medications, such as antibiotic eye-drops or pain killers, were prescribed if necessary.

### 2.2. Patient Assessments

Patients scheduled for glaucoma treatment with EyeOP-1 were evaluated at baseline and on the day of the treatment. Follow-up visits were performed after the procedure (Day 1 (D1), Month 1 (M1), Month 3 (M3), Month 6 (M6), Month 12 (M12), Month 18 (M18) and Month 24 (M24)) according to the World Glaucoma Association (WGA) recommendations [22].

IOP was measured using Goldmann Applanation Tonometry (GAT). Three IOP measurements were done on each occasion, and the average was used for the IOP evaluation. IOP measurements were masked to the surgeon.

The time of the day at which the measurements were taken was specified in the case report form. Measurements during follow-up visits were done at the same time as the enrolment visit ±4 h to minimize the effect of diurnal fluctuation. Lowering of IOP relative to the pre-operative value was assessed at each post-operative visit, and the final measurement was at 24 months.

Primary outcomes were complete success (defined as IOP lowering from baseline ≥20% without additional glaucoma medications) and vision-threatening complications. Secondary outcomes were the presence of complications and the reduction in the number of medications used. Efficacy parameters, success rate and safety parameters were recorded as follows.

#### 2.2.1. Efficacy Parameters:

- Mean IOP (mmHg) at each follow-up visit compared to baseline IOP;
- Mean IOP variation compared to baseline (%) at each follow-up visit;
- Number of ocular hypotensive medications at each follow-up visit.

#### 2.2.2. Success Rate:

- Complete success: IOP lowering >20% (<21 mmHg), and without supplemental glaucoma medication compared to baseline;
- Qualified success: 6 < IOP < 21 mmHg (IOP lowering < 20%) and reduction of glaucoma medications compared to baseline.

#### 2.2.3. Safety Parameters:

- Rate of per-operative device and/or procedure-related adverse events;
- Rate of post-operative device and/or procedure-related complications and adverse events at each follow-up visit;
- Best corrected visual acuity (BCVA) scored with reference to Logarithm of the Minimum Angle of Resolution (LogMAR).

### 2.3. Statistical Analysis

According to the planned analysis, all patients who gave informed consent to participate in the study, for whom the procedure associated with the treatment evaluation took place and for whom the inclusion and exclusion criteria were fulfilled at the baseline visit, were included in the data analyses.

Analysis of the outcomes of efficacy and safety were conducted overall and for each follow-up time point. Intraocular pressure was analyzed by the number and percentage of patients attaining the success criteria, together with the bilateral 5% confidence interval (CI). The mean and 95% CI of the change from baseline in IOP was computed. Survival curves (e.g., Kaplan-Meier) were used to evaluate the treatment success over time. Specific IOP results are presented graphically with scatter plots, as pre-treatment, baseline IOP (x-axis) versus post-treatment IOP (y-axis).

A complete analysis of adverse events included all the possible patient-related and device-related adverse events. Analysis included the incidence rate (number of patients with at least one adverse event) and the number of adverse events presented.

## 3. Results

Overall, 73 eyes of 66 patients were enrolled. Seven patients had protocol deviations and were excluded from the analysis: two patients had baseline IOP > 30 mmHg, four patients had previous glaucoma surgery, and one patient was aphakic. A total of 66 eyes of 59 patients (mean ± SD age 70.4 ± 11.4 years) were included in the final analysis. Baseline population characteristics are summarized in Table 1.

**Figure 1:**
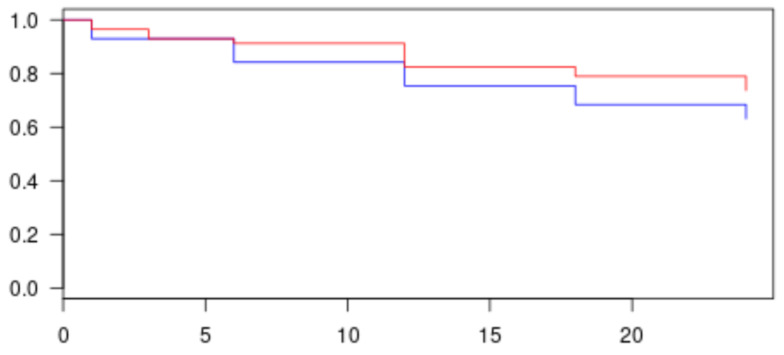
Kaplan–Meier graph of complete and qualified success in all population. Red line represents success patients at 24 months (complete and qualified); more than 74% of patients reached success. Blue line represents complete success patients only; more than 63% of success was obtained at 24 months.

In the intention to treat analysis, a 33% decrease in IOP was observed at 24 months with a success rate of 74%, of which 63% were complete success (Table 2 and Figure 1).

The lowering of IOP was statistically significant at each follow up visit (Table 2). In the sub-group of patients who reached the success criteria at M24 (per protocol analysis), the percent decrease in IOP (36%) was greater than the overall population with a maximum at the D1, M1 and M3 visits (D1 = 46%; M1 = 38%; M3 = 40%). There was no change in the number of glaucoma medications between baseline and 2 years post-operatively.

A total of 50 patients were evaluated at the 2-year visit. Among the sixteen others, during the follow-up period, filtering surgery was performed for seven patients due to uncontrolled IOP (10%), five patients died (not related to glaucoma disease), two patients decided to withdraw from the study, and two patients were lost to follow-up.

Perioperatively, three patients (5%) experienced pain during ultrasound activation. None of the patients experienced pain at the end of the UCP procedure. No serious adverse events related to the procedure or chronic hypotony and phthisis were reported. Locally, the most frequent complications were anterior chamber inflammation (<1 month) (62% of patients), conjunctival hyperemia (38%), transient mild mydriasis (19%), pupil deformation (7%) and transient hypotony (IOP < 6 mmHg) (5%) (Table 3). The presence of scleral marks was observed in 24/66 eyes (36%) during follow-up.

Baseline BCVA is shown in Table 4.

After 24 months, 43 patients were evaluated: 7 patients underwent filtering surgery before M24, 5 patients died, 2 patients were lost to follow up and 2 patients withdrew. A further seven subjects were excluded from the BCVA due to cataract surgery performed during the follow-up period, before M24 (five patients) and ocular adverse events not related to HIFU treatment (one corneal abrasion with previous band keratopathy and one central retinal vein occlusion at M24).

Mean BCVA was 0.40 ± 0.82 LogMAR in the overall population (Table 4). The change in visual function during follow-up in the group with BCVA ≤1 LogMAR was greatest 1 month after treatment (Table 4), after which time visual acuity improved and remained unchanged compared to baseline in 78% of patients at the end of follow-up, with four patients (10%) exhibiting a one line decrease and five patients (12%) exhibiting a decrease of two lines or more at M24. Among these latter five patients, four patients developed or had a worsening of a pre-existing cataract during follow-up, and one patient had superficial punctate keratitis in a pre-existing corneal disease.

## 4. Discussion

The UCP procedure with the EyeOP-1 in surgery-naïve glaucoma patients demonstrated a good safety and efficacy profile at M24. After treatment, patients benefited from a significant IOP lowering (33%) without serious complications related to the UCP procedure. Two patients developed vision loss, one for glaucoma progression and the other for central retinal vein occlusion at month 24; both were not considered to be related to the treatment.

The results of our multicenter study in terms of IOP lowering are in line with previous reports on surgery-naïve patients. In our population, the mean ± SD baseline IOP was 24.3 ± 2.9 mmHg, and 24 months after, at the last follow up visit, the mean ± SD IOP was 15.9 ± 3.6 mmHg. Previous published literature has reported a lowering percentage ranging from 28% to 38% after 1 year [20,21], from 40% to 42% after 2 years [23,24,25], and of 33% at 3 years [26]. In particular, in our study, the IOP decrease was maximal at 1 to 3 months after the treatment and then tended to stabilize.

The possibility of retreating patients after a first UCP procedure with greater lowering in IOP has also been described [23,27,28], but in our study, patients were not retreated, except for a patient who experienced an IOP spike at M3 and glaucoma progression until vision loss. The patient experienced pseudoexfoliative glaucoma with advanced damage (Cup/Disk ratio 0.9). For this patient, IOP decreased from 18 mmHg at D1 to 13 mmHg at M3, and it subsequently increased to 30 mmHg at M10 on maximum medical therapy. A second UCP was performed, and IOP decreased to 20 mmHg at D1, M1 and M6, but, unfortunately, the patient experienced vision loss from M1. The worsening of visual acuity in this patient did not appear to be related to the UCP procedure but could have been due to the advanced stage of glaucoma. Recently, Aptel [28] published a report of the efficacy and safety of repeated UCP in a cohort of 141 patients divided into early (IOP decrease <20% compared with baseline at 3 and/or 6 months) and late IOP increase (IOP decrease <20% compared with baseline after the first 6 months of follow up). In the first group, a mean IOP lowering of 34% (from 29.8 ± 8.2 to 18.5 ± 7.4 mmHg) was observed; in the second group (late IOP increase), the observed IOP lowering was even greater (43%) (from 31.9 ± 6.6 to 16.2 ± 5.2 mmHg). These results confirmed those obtained by previous authors both in terms of efficacy and, especially, safety of a second treatment.

The mean number of medications was similar from baseline until the last follow up visit, and, again, these findings agreed with previous reports [19,20,21,22,23,24,25,26,27,28].

In our study, 5% of patients complained of pain during the procedure. An explanation for this could be non-optimal anesthesia, but all the patients completed the treatment. The main adverse events reported were anterior chamber inflammation, conjunctival hyperemia, transient mild mydriasis, pupil deformation and transient hypotony. All these events were mild, transient and resolved spontaneously or with medications. Transient hypotony was observed in three patients, with complete resolution after 1 month in two patients and after 6 months in the other patient, without the need for additional medications. Mydriasis was verified in seven patients and resolved spontaneously after 6 months. Scleral marks were observed in 24/66 eyes during follow-up (36%), although this was not considered to be a complication and has been reported in literature without signs of inflammation or scleral protrusion [16,23,27]. Using OCT, a transient scleral thickening that reduced spontaneously to its initial value has been described [29]. Particularly, patients in the early post-operative period may also experience a less light-reactive pupil, sometimes associated with loss of accommodation, which seemed to normalize after 3–6 months [30,31]. Corneal edema observed in our population occurred at D1-D7 in an elderly patient (77 years of age) with previous corneal disease and on maximum hypotensive medical therapy (mean IOP 22 mmHg on four medications).

Macular edema was observed in two patients. The first case occurred at M1 and M3 and resolved at M6 after medical treatment and intravitreal injection of triamcinolone acetonide; the second case occurred 6 months after UCP in a patient with history of CME in the contralateral eye and was treated with two intravitreal injections of ranibizumab. This late case resolved 18 months post-operatively and was not considered to be related to the UCP procedure. Another patient presented central retinal vein occlusion at M24, and IOP was 20 mmHg under medications. This patient was treated by an intravitreal injection of dexamethasone using a drug delivery system at M25, followed by panretinal photocoagulation. This complication also was not considered related to UCP treatment.

Severe complications such as chronic hypotony, phthisis bulbi, suprachoroidal hemorrhage and retinal detachment, that have been reported with previous cyclodestructive procedures, were not observed after UCP treatment with the EyeOP-1 device [20]. We observed three cases of transient hypotony (IOP < 6 mmHg) (at D1, M1 and M3), but without choroidal detachment. There are also reports in literature of transient choroidal detachment after UCP that resolved after oral steroids [32]. Two patients in our study had vision loss, one for central retinal vein occlusion at M24 and one for glaucoma progression, but neither case was directly related to the UCP treatment.

An important consideration is that visual acuity remained unchanged in 78% of patients after 24 months of follow up in our study. This finding confirms the good safety profile of this technique. As previously described, cataract was the principal cause of decreased vision, and in four patients there was an evolution of pre-existing lens opacities.

There is published evidence of the possibility of retreating patients with early and late IOP increase with a second UCP procedure to achieve a greater IOP decrease while maintaining the same safety profile as the first procedure [23,27,28]. Additionally, a new version of the probe is now available, allowing an increased circumference of treated ciliary body (eight or ten instead of six sectors treated); this device achieved significant IOP control with a reduction in medication number in the Chinese population [33,34]. Another option that has been evaluated with encouraging results is association of UCP and phacoemulsification in the case of coexisting cataract with significant lowering of IOP and reduction in antiglaucoma medications [35].

Limitations of our study include the relatively small and non-homogeneous sample (patients were not stratified for the severity of glaucoma). Furthermore, patients were not randomized, and no subgroup analysis was performed since only 11 patients had exfoliative glaucoma and 1 patient had a pigmentary glaucoma. Another important consideration is that the lowering of IOP with UCP should be around 33% to 36%, and this represents a crucial point for patient selection; patients that need a lower target IOP may be not the best candidates for a single UCP procedure.

## 5. Conclusions

In conclusion, this 2-year report of safety and efficacy confirms the possibility of treating surgery-naïve glaucoma patients not only with drugs and conventional surgery but also with UCP.

## Figures and Tables

**Table 1:** Baseline demographic characteristics of eyes undergoing Ultrasound Cyclo Plasty (*n* = 66).

**Eyes** (Right/Left)	66 (37/29)
**Age (years), Mean (SD) (range)**	70.4 ± 11.4 (42–90)
**Gender** (male/female)	32/34 (48%/52%)
**Lens status**	
Phakic	37 (56%)
Pseudophakic	29 (44%)
**Type of glaucoma**	
Primary open-angle glaucoma	54 (82%)
Exfoliative glaucoma	11 (16%)
Pigmentary glaucoma	1 (2%)
**Previous ocular treatments (total number of procedures)**	
Incisional surgery (trabeculectomy)	0
Cyclodestructive procedure	0
Laser trabeculoplasty (SLT/ALT)	10 (15%)
**Mean preoperative IOP**	24.3 ± 2.9 [21,22,23,24,25,26,27,28,29,30]
**Pre-operative glaucoma medications**	
Eyedrops	2.3 ± 1.1
Tablets (Acetazolamide)	8/66

IOP: intraocular pressure.

**Table 2:** Intraocular pressure, glaucoma medication and success rate during follow-up with number of glaucoma medications in the overall population (A) and in patients with success at Month 24 (B). (Efficacy results).

Overall Population (A)			Success at Month-24 (B)		
	Mean IOP (No. Patients) (Eyedrops */Tablets **)	Relative IOP Reduction (%)	Success Rate (%)	Mean IOP (No. Patients) (Eyedrops */Tablets **)	Relative IOP Reduction (%)
Baseline	**24.3** ± 2.9 (66) (2.3 ± 1.1/8)	NA	**NA**	**24.0** ± 2.9 (42) (2.4 ± 1.3/4)	NA
Day 1	**13.6** ± 4,9 (65) (2.3 ± 1.1/9)	44%	94%	**12.9** ± 3,9 (41) (2.4 ± 1.3/4)	46%
Month 1	**16.2** ± 5.1 (65) (2.2 ± 1.1/6)	33%	83%	**14.8** ± 4.3 (41) (2.3 ± 1.3/3)	38%
Month 3	**16.0** ± 4.8 (66) (2.3 ± 1.1/5)	34%	83%	**14.5** ± 3.7 (42) (2.3 ± 1.4/3)	40%
Month 6	**16.8** ± 5.2 (64) (2.2 ± 1.0/6)	31%	85%	**16.3** ± 4.8 (42) (2.1 ± 1.3/3)	33%
Month 12	**16.5** ± 4.3 (60) (2.2 ± 1.0/3)	32%	80%	**16.0** ± 4.0 (42) (2.2 ± 1.2/2)	34%
Month 18	**16.3** ± 4.4 (54) (2.1 ± 1.0/2)	32%	77%	**15.8** ± 4.4 (40) (2.1 ± 1.2/2)	34%
Month 24	**15.9** ± 3.6 (50) (2.2 ± 1.0/3)	33%	77%	**15.4** ± 3.6 (42) (2.2 ± 1.2/2)	36%

* Mean number of hypotensive eyedrops; ** number of patients with tablets (Acetazolamide); D1/M1: one visit not done; M6: two patients discontinued (filtering surgery); M12: four patients discontinued (filtering surgery), one withdrawal (patient decision), one lost to follow-up; M18: six patients discontinued (filtering surgery), two visits not done, one withdrawal (patient decision), one lost to follow-up, two deaths; M24: seven patients discontinued (filtering surgery), two withdrawal (patient decision), two lost to follow-up, five deaths.

**Table 3:** Postoperative complications.

Postoperative Complications	*n* (%)
Anterior chamber inflammation (<1 month)	41 (62%)
Conjunctival hyperemia (<1 month)	25 (38%)
Transient mild mydriasis *	13 (19%)
Superficial punctate keratitis	10 (15%)
Pupil peak (pupil deformation)	5 (7%)
Transient hypotony (IOP < 6 mmHg)	3 (5%)
Ocular pain (<24 h)	2 (3%)
Macular edema	2 (3%)
Uveitis	1 (2%)
Subconjunctival hemorrhage	1 (2%)
Corneal edema	1 (2%)
Monocular double vision	1 (2%)

* Monocular double vision. Reported in one patient at M3 (BCVA = 20/20). No treatment, but new glasses. (At M1: no complaint (BCVA = 20/25); at M6: no complaint: BCVA = 20/20).

**Table 4:** Visual acuity at baseline and during follow-up.

LogMar Visual Acuity		Baseline	Month 1	Month 3	Month 6	Month 12	Month 18	Month 24
Overall population	*n*	66	65	66	63	58	48	43 *
	Mean ± SD	0.43 ± 0.81	0.50 ± 0.80	0.46 ± 0.81	0.48 ± 0.88	0.54 ± 0.93	0.57 ±1.00	0.40 ± 0.82
Vision Group (A) **	*n*	60	59	60	57	52	43	40
	Mean ± SD	0.19 ± 0.24	0.27 ± 0.28	0.23 ± 0.28	0.21 ± 0.31	0.28 ± 0.51	0.30 ±0.63	0.23 ± 0.46
	Unchanged	-	26 (44%)	40 (67%)	39 (69%)	35 (67%)	27 (63%)	31 (78%)
	Loss 1 line	-	21 (36%)	12 (20%)	13 (23%)	10 (19%)	11 (26%)	4 (10%)
	Loss ≥ 2 lines	-	12 (20%)	8 (13%)	5 (9%)	7 (14%)	5 (12%)	5 (12%)
Low vision Group (B) ***	*n*	6	6	6	6	6	5	3
	Mean ± SD	2.80 ± 0.59	2.80 ± 0.59	2.80 ± 0.59	3.02 ± 0.24	2.85 ±0.48	2.84 ±0.54	2.80 ± 0.75
	Unchanged	-	6 (100%)	6 (100%)	4 (67%)	5 (83%)	4 (80%)	2 (67%)
	Loss 1 line	-	-	-	1 (16.5%)	-	-	-
	Loss ≥ 2 lines	-	-	-	1 (16.5%)	1 (17%)	1 (20%)	1 (33%)

* see note 1; ** Visual acuity ≤1 LogMar (A); *** Visual acuity ≥1.7 LogMar (B); Note 1. Loss ≥2 lines. In vision group (A) (*n* = 5): progression of pre-existing cataract (*n* = 4), unexplainable vision loss (possible glaucoma progression) (*n* = 1); in low vision group (B) (*n* = 1): myopic patient glaucomatous damage associated with progressive myopic macular dystrophy (n = 1).

## Manufacturer Name

EYE TECH CARE—Rillieux-La-Pape—France, owner of the EyeOP-1.
